# Permeability-Selectivity Analysis of Microfiltration and Ultrafiltration Membranes: Effect of Pore Size and Shape Distribution and Membrane Stretching

**DOI:** 10.3390/membranes6030040

**Published:** 2016-08-06

**Authors:** Muhammad Usama Siddiqui, Abul Fazal Muhammad Arif, Salem Bashmal

**Affiliations:** Mechanical Engineering Department, King Fahd University of Petroleum & Minerals, Dhahran 31261, Saudi Arabia; musiddiqui@gmail.com (M.U.S.); bashmal@kfupm.edu.sa (S.B.)

**Keywords:** permeability-selectivity, membrane stretching

## Abstract

We present a modeling approach to determine the permeability-selectivity tradeoff for microfiltration and ultrafiltration membranes with a distribution of pore sizes and pore shapes. Using the formulated permeability-selectivity model, the effect of pore aspect ratio and pore size distribution on the permeability-selectivity tradeoff of the membrane is analyzed. A finite element model is developed to study the effect of membrane stretching on the distribution of pore sizes and shapes in the stretched membrane. The effect of membrane stretching on the permeability-selectivity tradeoff of membranes is also analyzed. The results show that increasing pore aspect ratio improves membrane performance while increasing the width of pore size distribution deteriorates the performance. It was also found that the effect of membrane stretching on the permeability-selectivity tradeoff is greatly affected by the uniformity of pore distribution in the membrane. Stretching showed a positive shift in the permeability-selectivity tradeoff curve of membranes with well-dispersed pores while in the case of pore clustering, a negative shift in the permeability-selectivity tradeoff curve was observed.

## 1. Introduction

Microfiltration and ultrafiltration are widely used techniques in applications ranging from wastewater treatment to biomedical applications and the food industry. In water desalination, for example, the use of ultrafiltration membranes instead of conventional pre-treatment improves the Reverse Osmosis (RO) feed quality and provide stable permeability.

**Figure 1:**
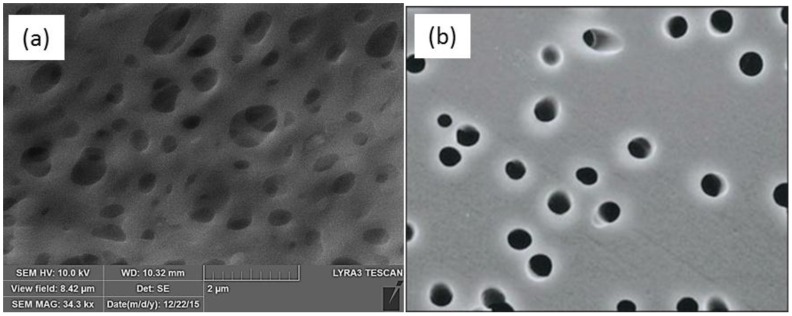
Microstructure of an (**a**) phase-inversion PES membrane and (**b**) track-etched membrane [1].

Microfiltration and ultrafiltration membranes are generally made using organic polymers, such as polytetrafluoroethylene (PTFE), polyethylene terephthalate (PET), polyvinylidene fluoride (PVDF), polypropylene (PP), polyethylene (PE), polysulfone (PS), and polyether sulfone (PES) and are prepared by techniques, such as track-etching, stretching, and phase inversion. The microstructures of phase-inversion and track-etched membranes are shown in Figure 1.

Microfiltration and ultrafiltration are pressure driven processes that work by removing particles larger than the pore size of the membrane through a sieving mechanism. The quality of separation is normally expressed by the rejection factor or the separation factor of the membrane for a given solute. The rejection factor is defined as 1 − *c_perm_/c_feed_* while the separation factor is defined as *c_feed_/c_perm_* (where *c_perm_* = permeate concentration and *c_feed_* = feed concentration). Ideally, a membrane should combine high permeability and high rejection rate. Practically, this is not the case as permeability and rejection rate cannot both be increased at the same time [2].

In order to understand and predict the performance of microfiltration and ultrafiltration membranes, several efforts have been made to formulate models for the membrane performance under the influence of fouling [3,4,5,6], pore size distribution [2,7], and pore shape [8,9]. Variation in pore sizes has been found to affect membrane performance significantly. Effect of pore size distribution in track-etched membranes on the permeability-selectivity characteristics of ultrafiltration membranes was studied by Mehta and Zydney [2] for circular pores and by Kanani et al. [9] for slot-shaped pores. Kanani et al. also found that using slot-shaped pores (very high aspect ratio) resulted in higher permeate flux compared to circular pores for same rejection ratios. Increased aspect ratio has also been linked to reduced fouling rates in membranes [10].

To take advantage of improved membrane performance when pore aspect ratio is high, studies have also been conducted to see the effect of artificially increasing pore aspect ratio by uniaxial stretching of the membranes [11,12,13]. Morehouse et al. [11,12] and Worrel [13] carried out experimental and numerical studies to study the effect of uniaxial stretching on the pore geometry and performance of PET track-etched membranes and phase-inversion PES and PVDF membranes, respectively. Both found that an increase in the pore aspect ratio resulted in improved permeate flux, but no definite trend in the rejection rate of the solutes was observed as the uniaxial strain applied to the membrane was increased.

Although models of membrane performance that take into account pore size distribution have been published for various pore geometries, they are not applicable to real membranes. As is evident from Figure 1, conventional membrane preparation methods do not result in well-defined pore shapes. For example, the track-etched membrane in Figure 1b, not only has a pore size distribution but also a pore aspect ratio distribution. Similarly, the process of membrane stretching will also introduce an aspect ratio distribution in the membrane. Currently, no model is available that can predict the permeability-selectivity characteristics of microfiltration and ultrafiltration membranes taking into account the pore size, as well as pore aspect ratio distributions.

In the current work, three major tasks are carried out. First, a model to study permeability-selectivity trade-off of membranes with pore size and aspect ratio distributions is formulated and is used to study the effect of aspect ratio and size distribution on the permeability-selectivity trade-off of the membrane. Second, a finite element model for porous membranes is developed and is used to study the effect of membrane stretching on pore size and aspect ratio distributions and on the permeability-selectivity trade-off. Finally, the effect of porosity dispersion uniformity on the performance of stretched membrane is studied.

## 2. Permeability-Selectivity Analysis

A methodology to carry out permeability-selectivity analysis of ultrafiltration and microfiltration membranes has been presented by Mehta and Zydney [2] for membranes with circular pores of variable sizes and later extended by Kanani et al. [9] for slot pores. The following development for membranes with circular pores was done by Mehta and Zydney [2] to determine the permeability of the solvent through a membrane with circular pores and is presented here for the sake of completeness with some additional intermediate steps. The methodology is then extended to include the effect of pore shape distribution.

### 2.1. Circular Pores with Size Distribution

Under the assumption of a convection dominated process, the velocity of all impurities in the feed is the same as the solvent and the separation coefficient of the membrane for the large solute to be filtered is defined as:

(1) $$\alpha =\frac{S_{small}}{S_{large}}$$

where $S_{large}$ is the selectivity of the larger solute that needs to be separated and $S_{small}$ is the selectivity of the small solutes that pass through the membrane without any hindrance.

The selectivity of the larger solute that needs to be filtered can be determined using the expression developed by Zeman and Wales [14]:

(2) $$S_{a}\left(r\right)={\left(1-\lambda \right)}^{2}\left(2-{\left(1-\lambda \right)}^{2}\right)\exp\left(-0.7146\lambda^{2}\right)$$

where $\lambda =r_{s}/r$, *r*, and $r_{s}$ are the pore and solute radii.

The permeability of the solvent through the membrane can be determined by assuming that the pores are perfect cylinders. Under this condition, the Hagen–Poiseuille equation is valid. The volumetric flow rate of the solvent through the membrane is:

(3) $$Q=\frac{\Delta P}{8\mu \delta_{m}}N_{p}\pi r^{4}$$

where *Q* is the flow rate (m^3^/s), Δ*P* is the pressure drop (Pa) across the membrane, μ is the viscosity (Pa∙s) of the solvent, δ*_m_* is the membrane thickness (m), and *N_p_* is the number of pores in the membrane. The volumetric flux through the membrane becomes:

(4) $$J_{v}=Q/A_{mem}$$

where $A_{mem}$ is the membrane area defined by:

(5) $$A_{mem}=\frac{N_{p}\pi r^{2}}{\varphi }$$

and ϕ is the porosity fraction in the membrane.

Using Equations (3)–(5), the permeability of the solvent through the membrane can be defined using:

(6) $$L_{p}=\frac{J_{v}}{\Delta P}=\frac{\varphi r^{2}}{8\mu \delta_{m}}$$

Equations (2) and (6) can be used to generate the permeability-selectivity curves for membranes. The effect of variation in the pore radius can also be included in the equations for permeability and selectivity. This is done by averaging the permeability and selectivity over the entire range of pore radii. Mochizuki and Zydney [7] carried out a theoretical analysis to determine the average permeability and selectivity of membranes. The average permeability and selectivity are given by Equations (7) and (8), respectively:

(7) $${\bar{L}}_{p}=\frac{\varphi }{8\mu \delta_{m}}\frac{\int_{0}^{\infty }n\left(r\right)r^{4}dr}{\int_{0}^{\infty }n\left(r\right)r^{2}dr}$$

(8) $${\bar{S}}_{a}=\frac{\int_{0}^{\infty }S_{a}\left(r\right)n\left(r\right)r^{4}dr}{\int_{0}^{\infty }n\left(r\right)r^{4}dr}$$

where *n*(*r*) is the probability density function of pore radii.

### 2.2. Elliptical Pores with Size and Aspect Ratio Distribution

Using a similar approach to Mehta and Zydney [2], the permeability of a solvent through a membrane with elliptical pores was derived. The solution of the Hagen–Poiseuille equation for elliptical cross-section leads to the following equation for solvent flow rate:

(9) $$Q=\frac{N_{p}\Delta P}{4\mu \delta_{m}}\frac{\pi a^{3}b^{3}}{a^{2}+b^{2}}$$

where *a* and *b* are the major and minor axes half lengths of the elliptical cross section of the pore.

The permeability of the solvent through the membrane is, therefore:

(10) $$L_{p}=\frac{\varphi }{4\mu \delta_{m}}\frac{a^{2}b^{2}}{a^{2}+b^{2}}$$

To take into account the variation in the pore sizes in the membrane, the area weighted average of Equation (10) is taken. The average permeability through the membrane is given by Equation (11):

(11) $${\bar{L}}_{p}=\frac{\varphi }{4\mu \delta_{m}}\frac{\int_{0}^{\infty }\int_{0}^{\infty }n\left(a\right)n\left(b\right)\frac{a^{3}b^{3}}{a^{2}+b^{2}}dadb}{\int_{0}^{\infty }\int_{0}^{\infty }n\left(a\right)n\left(b\right)ab\;dadb}$$

where *n*(*a*) and *n*(*b*) are the probability density functions of the major and minor axes’ half lengths of the pores.

To determine the average selectivity of the membrane, it is noted that the selectivity will depend on the minor axis length of the pore cross section. Setting $\lambda =r_{s}/b$ and assuming Equation (2) is still valid for determining selectivity, the average selectivity of membranes with elliptical pores of variable sizes can be determined using:

(12) $${\bar{S}}_{a}=\frac{\int_{0}^{\infty }S_{a}\left(b\right)n\left(b\right)b^{2}db}{\int_{0}^{\infty }n\left(b\right)b^{2}db}$$

## 3. Effect of Pore Geometry on Membrane Performance

In order to study the effect of pore aspect ratio on the permeability-selectivity performance of microfiltration and ultrafiltration membranes, a study was conducted in which the aspect ratio of the pores was changed while keeping the pore cross-section area constant. The parameters used in the model were solute radius *r_a_* = 3.65 nm for bovine serum albumin protein, membrane porosity fraction ϕ *=* 0.3, membrane thickness δ *=* 0.3 μm and solvent viscosity μ *=* 0.001 Pa∙s. The log-normal probability distribution function, given by Equation (13) was used to describe the pore size variation within the membrane [7].

(13) $$n\left(x\right)=\frac{n_{0}}{x\sqrt{2\pi }}{\left[\ln\left(1+{\left(\sigma /\bar{x}\right)}^{2}\right)\right]}^{-1/2}\exp\left\{-{\frac{\left(\ln\left(x/\bar{x}\right){\left[1+{\left(\sigma /\bar{x}\right)}^{2}\right]}^{1/2}\right)}{2\ln\left[1+{\left(\sigma /\bar{x}\right)}^{2}\right]}}^{2}\right\}$$

where σ is the standard deviation of *x*, $\bar{x}$ is the mean value of *x*, and *n*_0_ is the maximum possible value of *n*(*x*). The two parameters that control the distribution are mean $\bar{x}$ and the normalized standard deviation $\sigma /\bar{x}$.

**Figure 2:**
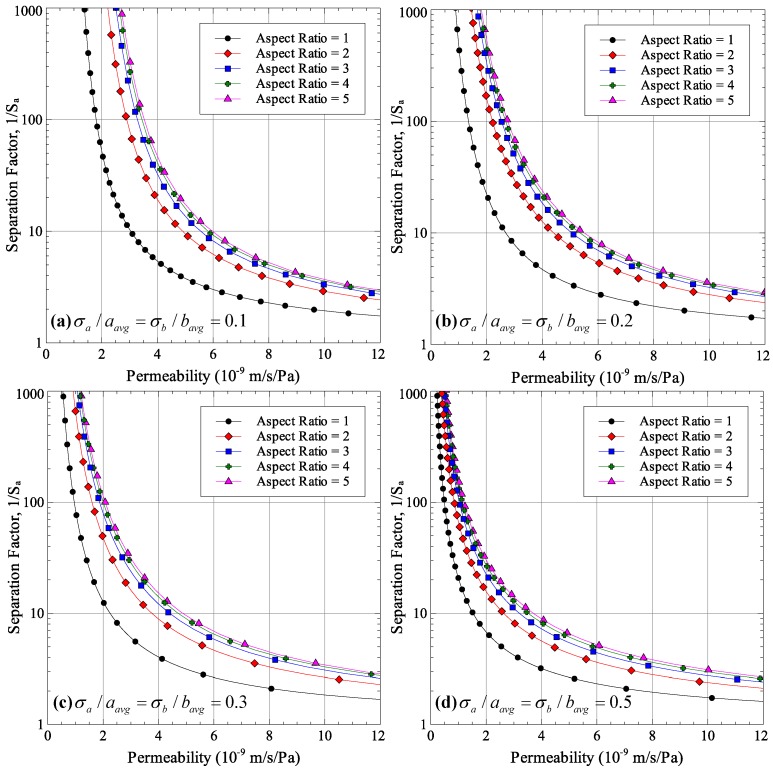
Effect of pore aspect ratio on permeability-selectivity trade-off.

**Figure 3:**
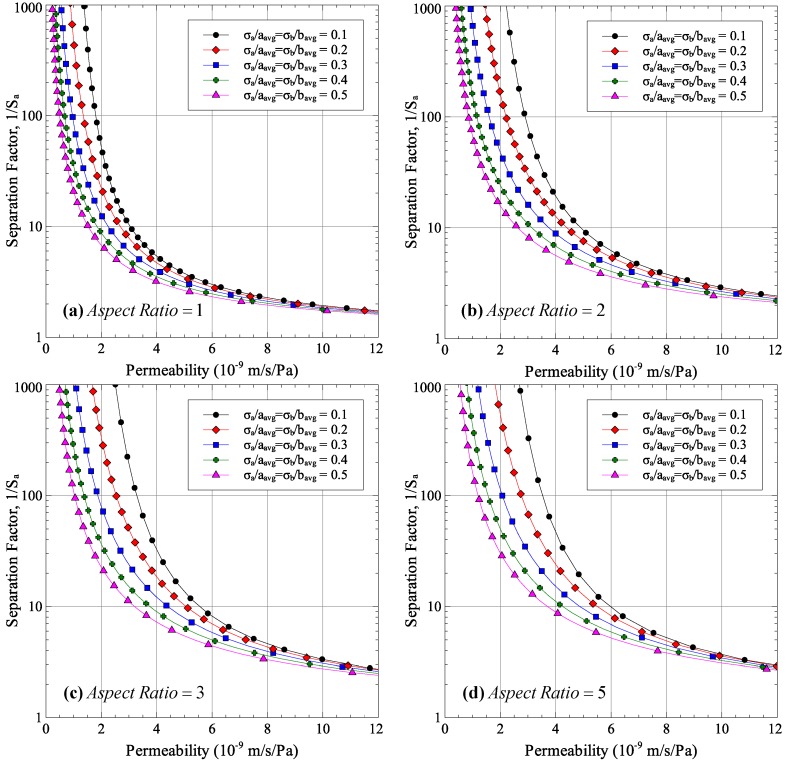
Effect of pore size distribution on permeability-selectivity trade-off.

Two studies were conducted; first, to see the effect of average aspect ratio on permeability-selectivity trade-off for different normalized standard deviations of pore sizes *a* and *b*, and second, to see the effect of normalized standard deviations of pore sizes *a* and *b* on the performance for different aspect ratios. The results are shown in Figure 2 and Figure 3. Each point in the figures was generated by selecting an average pore radius *r*, calculating the average major and minor axes half lengths under the condition that the pore area remains the same for all aspect ratios and calculating permeability and selectivity using Equations (11) and (12), respectively.

As can be seen from Figure 2, increasing the average aspect ratio improves the performance of microfiltration and ultrafiltration membranes for all size distributions. In other words, for the same separation factor, a higher permeate flux can be obtained if the pore aspect ratio is increased. This result agrees with the experimental findings of Worrel [13] and Morehouse et al. [11,12] who found the same effect of increasing aspect ratio of permeate flux. This strengthens confidence in the theoretical development carried out in the current work.

The effect of pore size distribution on the membrane performance is shown in Figure 3. The figure shows the detrimental effect of non-uniform pore size on the permeability-selectivity trade-off curve. Having a larger standard deviation of pore sizes results in a lower separation factor for all aspect ratios. In order to achieve the same separation factor, the pore size needs to be reduced, which reduces the permeability.

## 4. Membrane Stretching

Previous studies show that membrane stretching can have a positive effect on membrane permeability [11,12,13]. On the other hand, the effect of stretching on the separation factor could not be properly identified as experiments showed that there were cases in which the separation factor increased, remained unchanged, or even reduced [13].

In the current work, a finite element model of a microfiltration membrane was developed to analyze the effect of membrane stretching on the size and shape distributions of pores in the stretched membrane. Using the results of the finite element model, the effect of membrane stretching on the permeability-selectivity trade-off curve was also analyzed. This section presents the development of the finite element model and the resulting pore sizes due to membrane stretching. The effect of membrane stretching on its performance is analyzed in the next section.

### 4.1. Finite Element Model for Membrane Stretching

**Figure 4:**
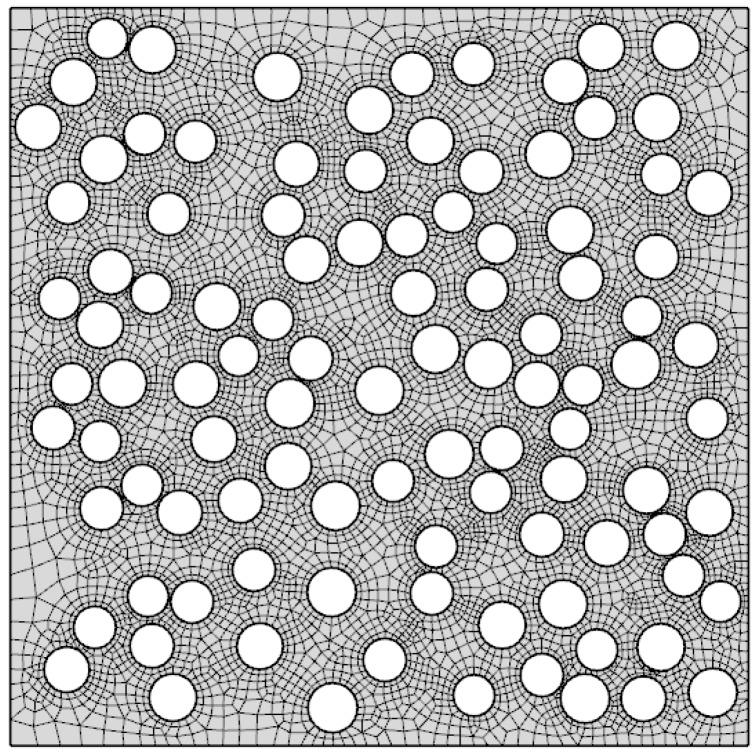
Membrane geometry with finite element mesh.

The membrane stretching problem consists of a finite element model of the representative volume element (RVE) of the porous membrane. The constitutive behavior of the membrane material is defined explicitly as viscoelastic-rate-independent plastic. The geometry of a 30% porous membrane with an average pore size of 0.1 μm was generated using an in-house code and is shown in Figure 4 along with the finite element mesh. The microscale model is defined by Equations (14)–(17).

(14) $$-\nabla \cdot \left(1+\nabla u\right)S=F_{V}$$

(15) $$S=C:\varepsilon_{el}+S_{q}$$

(16) $$\varepsilon_{el}=\varepsilon -\varepsilon_{pl}$$

(17) $$\varepsilon =\frac{1}{2}\left[{\left(\nabla u\right)}^{T}+\nabla u+{\left(\nabla u\right)}^{T}\nabla u\right]$$

where *u* is the displacement field, *S* is the second Piola-Kirchhoff stress, *F_v_* is the body load, *C* is the elasticity tensor, *S_q_* is the relaxation stress due to viscoelasticity, and $\varepsilon ,\;\varepsilon_{el}\text{ and }\varepsilon_{pl}$ are the total, elastic, and plastic strain tensors.

For viscoelasticity, the bulk modulus was assumed to be constant while the shear modulus was assumed to be defined by the generalized Maxwell model. The model consists of several spring-damper branches in parallel each of which is defined by a shear modulus and a relaxation time. Considering *m* parallel branches, the viscoelasticity model can be described using Equations (18) and (19):

(18) $$S_{q}=\sum_{m}2G_{vm}\left(e_{el,dev}-\varepsilon_{vm}\right)$$

(19) $$\tau_{vm}{\dot{\varepsilon }}_{vm}+\varepsilon_{vm}=\varepsilon_{el,dev}$$

where *G_vm_* is the shear modulus in branch *m*, $\tau_{vm}$ is the relaxation time of branch *m*, $\varepsilon_{vm}$ is the viscoplastic strain of branch *m*, and $\varepsilon_{el,dev}$ is the deviatoric part of the elastic strain tensor.

The rate-independent plasticity of the membrane material was modeled using the bilinear isotropic hardening model. The model is described by Equations (20)–(23):

(20) $${\dot{\varepsilon }}_{p}={\dot{\varepsilon }}_{p,eff}\frac{\partial F}{\partial S}$$

(21) $$F=\sigma_{mises}-\sigma_{ys}$$

(22) $$\sigma_{ys}=\sigma_{y0}+\frac{E_{T,iso}}{1-\frac{E_{T,iso}}{E}}\varepsilon_{p,eff}$$

(23) $${\dot{\varepsilon }}_{p,eff}\geq 0,\;F\left(\sigma ,\sigma_{ys}\right)\leq 0,\;{\dot{\varepsilon }}_{p,eff}F=0$$

where $\varepsilon_{p}$ is the plastic strain tensor, $\varepsilon_{p,eff}$ is the von Mises effective plastic strain, $\sigma_{mises}$ is the von Mises stress, σ*_y_*_0_ and σ*_y_* are the initial and current yield stress, and *E* and *E_T_* are the elastic and tangential moduli.

**Figure 5:**
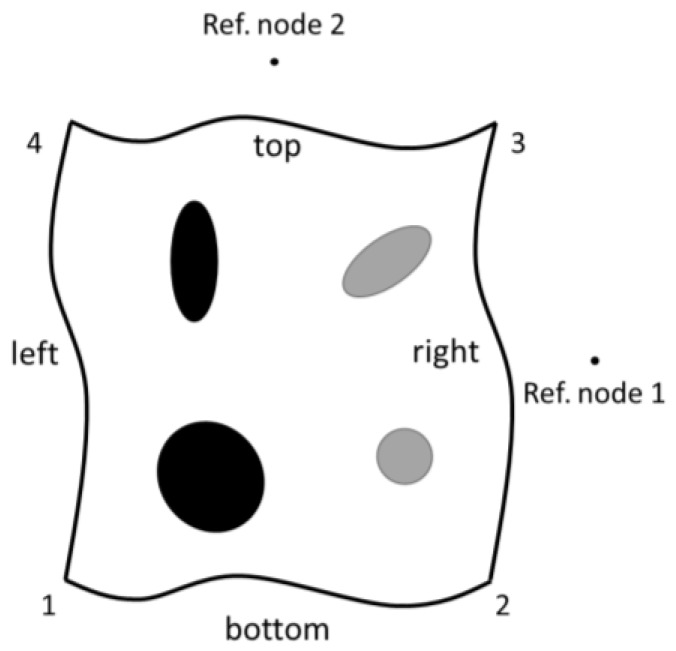
Applying periodic boundary conditions to a microscale RVE.

Since the finite element model represents a part of a larger membrane, periodic boundary conditions are applied to the model using constraint equations. For a two-dimensional model, shown in Figure 5, the constraint equations are given as Equations (24)–(28). Uniaxial stretching is applied to the membrane by controlling the displacement of reference node 1.

(24) $${\vec{u}}_{2}-{\vec{u}}_{1}-{\vec{u}}_{ref_{1}}=0$$

(25) $${\vec{u}}_{4}-{\vec{u}}_{1}-{\vec{u}}_{ref_{2}}=0$$

(26) $${\vec{u}}_{3}-{\vec{u}}_{1}-{\vec{u}}_{ref_{1}}-{\vec{u}}_{ref_{2}}=0$$

(27) $${\vec{u}}_{right,no\text{​ }edges}-{\vec{u}}_{left,no\text{​ }edges}-{\vec{u}}_{ref_{1}}=0$$

(28) $${\vec{u}}_{top,no\text{​ }edges}-{\vec{u}}_{bottom,no\text{​ }edges}-{\vec{u}}_{ref_{2}}=0$$

In the current work, the membrane material was assumed to be PET. The properties used in the current work were determined by Hanks et al. [15] who experimentally determined the stress-strain response and the viscoelastic material properties of dense PET. The properties used in the finite element model are listed in Table 1.

**Figure 6:**
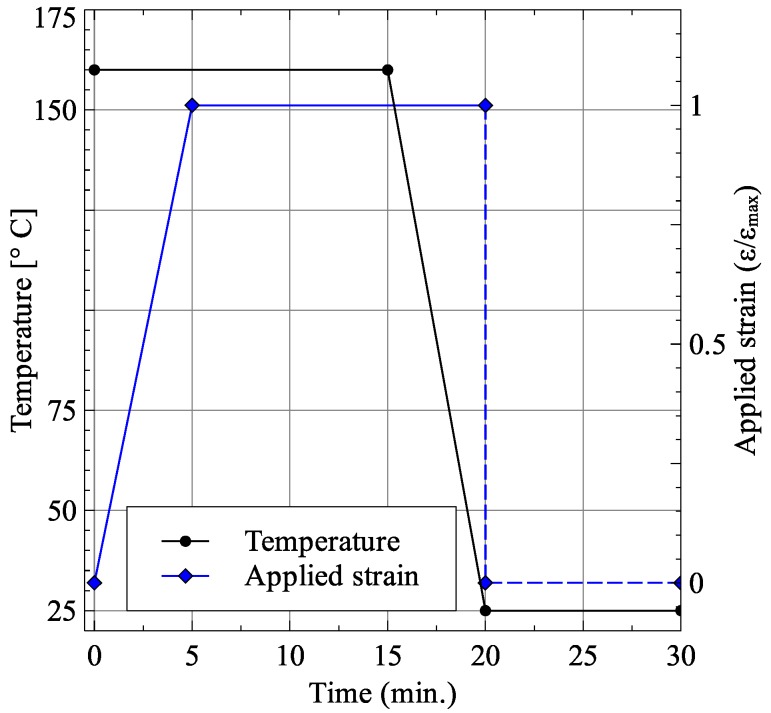
Applied temperature and strain load.

The stretching process was carried out at 160 °C by applying the total strain over a period of 5 min. This was followed by a holding time of 10 min to allow stress relaxation followed by cooling the membrane to room temperature over a period of five minutes. Finally, the applied strain load was released to remove any elastic strains within the membrane. The membrane temperature and applied strain load are shown in Figure 6.

### 4.2. Finite Element Modeling Results

**Figure 7:**
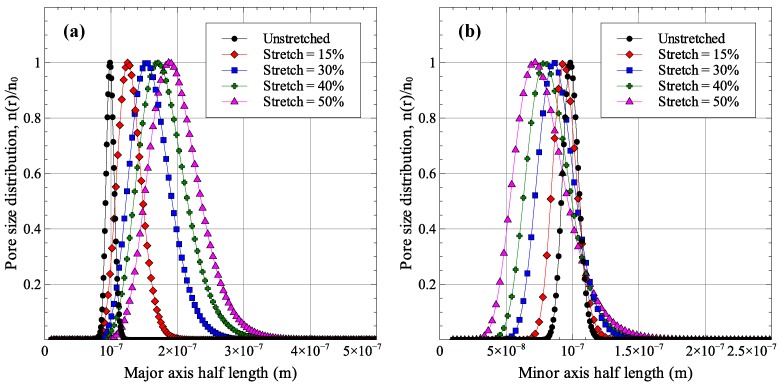
(**a**) Major and (**b**) minor axes size distribution for stretched membranes.

**Figure 8:**
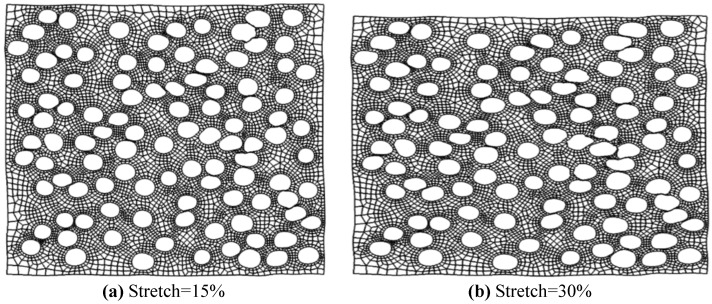
Deformed geometries of stretched membranes for (**a**) 15% stretch and (**b**) 30% stretch.

For the membrane geometry presented in Figure 4, the cases of 15%, 30%, 40%, and 50% uniaxial stretch were solved and analyzed. A summary of pore sizes after membrane stretching is presented in Table 2, while Figure 7 shows the distribution of major and minor axes sizes. The deformed geometries for two of the solved cases (15% and 30% stretch) are shown in Figure 8.

As expected, increasing the uniaxial strain applied to the membrane increases the pore aspect ratios. The average pore aspect ratio increases from 1.374 for 15% stretch to 2.732 for 50% stretch. The stretching process also affects the distribution of pore sizes as increasing stretching strains result in larger standard deviations in pore sizes. The maximum normalized standard deviation was observed for the 50% stretch case with values of 0.201 and 0.256 for major and minor axes sizes. As was shown in Figure 2 and Figure 3, increasing the aspect ratio improves the membrane performance while a wider distribution of pore sizes results in a degradation of membrane performance. Since membrane stretching results in an increase in pore aspect ratios, as well as the width of the distribution of sizes, stretching will not necessarily result in an improved permeability-selectivity trade-off.

## 5. Effect of Stretching on Membrane Performance

**Figure 9:**
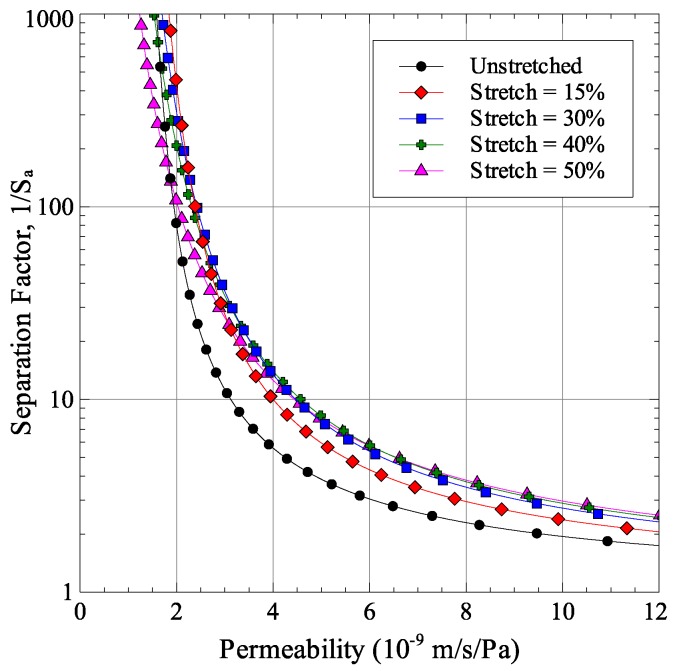
Effect of membrane stretching on permeability-selectivity trade-off.

Using the finite element model results of Section 4 and the permeability and selectivity models formulated in Section 2, the effect of membrane stretching on its permeability-selectivity trade-off was analyzed. As was discussed in Section 4.2 and shown in Figure 7, there are two competing effects of stretching that modify the membrane performance. First, stretching causes the pore aspect ratios to increase, which improves performance. Second, the width of pore size distribution is also increased, which degrades membrane performance. Their combined effect on membrane performance for the stretching cases considered in Section 4.2 is shown in Figure 9. Each point in the figure is generated by taking an initial pore radius *r* and applying the average major and minor axes stretches to it. The normalized standard deviation was assumed to be independent of the average pore size. The figure shows that the cases of 15% and 30% stretches show a positive effect of the permeability-selectivity trade-off curve. The performance starts to degrade at higher stretch magnitudes. This is easily observable at high selectivity values. The degradation of performance can be associated with the large width of pore size distribution which is numerically represented as the normalized standard deviation in Table 2.

### Effect of Porosity Dispersion Quality on Membrane Performance

The results in Figure 9 show that at higher stretch levels, the effect of size distribution dominates the effect of pore aspect ratio. This results in a worse performance than the case of a lower stretch. In this section, a study is presented to examine the hypothesis that the pore size distribution after stretching is related to the uniformity of pore dispersion.

**Figure 10:**
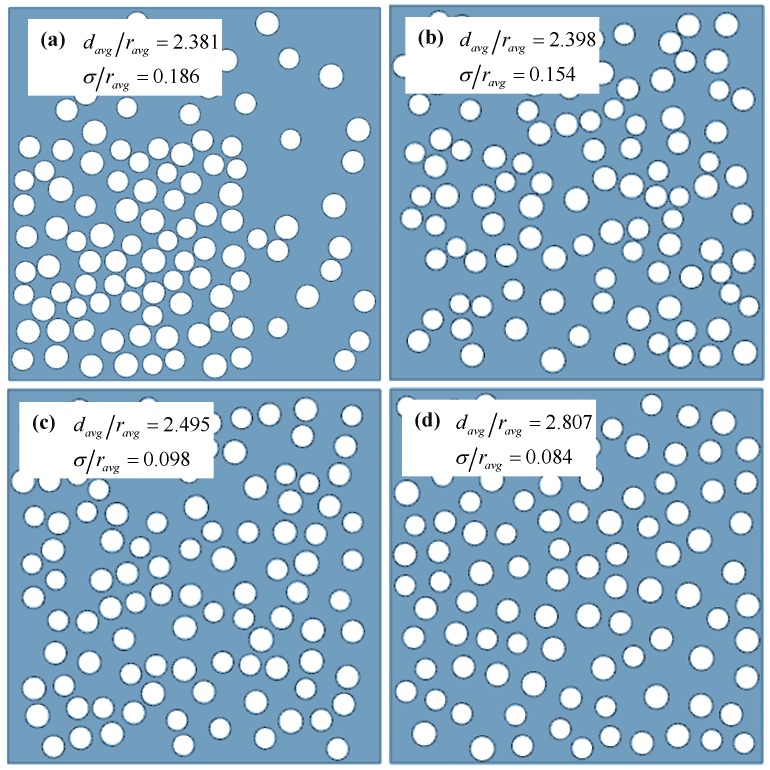
Porous membrane RVEs with controlled porosity dispersion.

**Figure 11:**
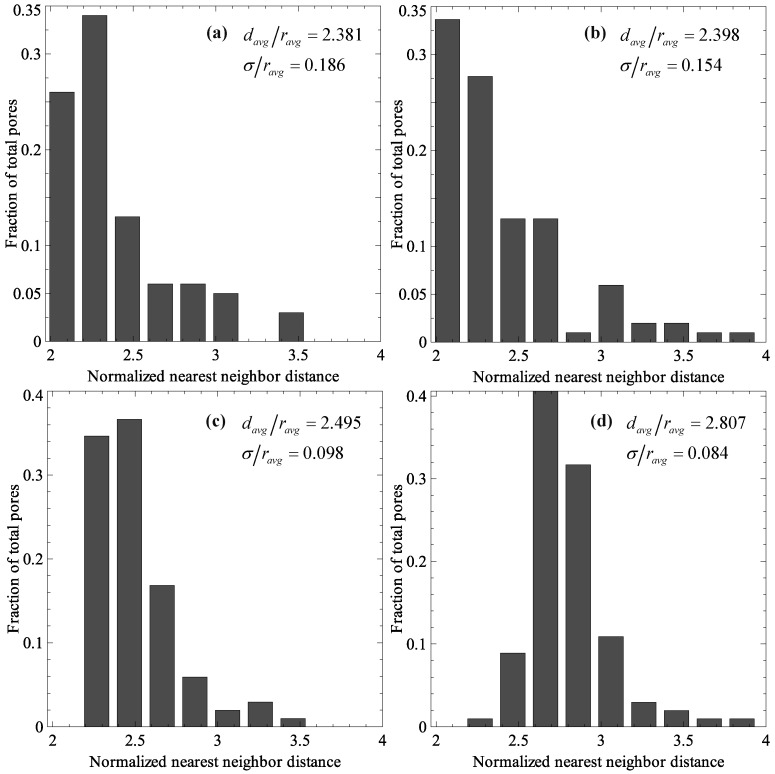
Normalized nearest neighbor distances in membrane RVEs with controlled porosity dispersion.

To start, four porous membrane RVEs were generated with different pore dispersion uniformities. The dispersion quality for each microstructure was quantified by first calculating the nearest-neighbor distances *d* (calculated from center to center) for all pores and representing them as the normalized average nearest-neighbor distance *d_avg_/r_avg_* and its standard deviation σ/*r_avg_*. A higher average nearest-neighbor distance and a lower standard deviation represent better dispersion. The four membrane RVEs, along with the dispersion quality parameters, are shown in Figure 10, in which case (b) is the one studied in previous sections. The dispersion quality is increasing from case (a) to (d). The distributions of normalized nearest neighbor distances in the four RVEs are shown in Figure 11.

**Figure 12:**
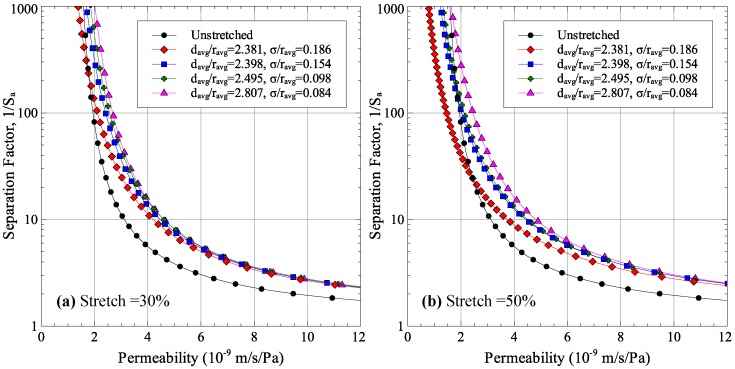
Effect of porosity dispersion on permeability-selectivity trade-off for (**a**) 30% stretch and (**b**) 50% stretch.

Using the four RVEs, the cases for 30% and 50% stretches were solved using finite element analysis and the permeability-selectivity trade-off curves were generated which are shown in Figure 12. The figure shows the significant effect that porosity dispersion quality can have on the performance of stretched membrane. As dispersion quality is improved, the selectivity of stretched membranes improves for the same permeability.

The results presented in this section are significant as they can help explain why previous experimental studies with stretched membranes showed that the separation factor increased, stayed the same, or even reduced for stretched membranes [13].

## 6. Conclusions

Permeability-selectivity tradeoff analysis provides a simple tool for the analysis and comparison of the performance of microfiltration and ultrafiltration membranes. In the current work, a model to carry out permeability-selectivity analysis is formulated that takes into consideration the distribution of pore sizes and aspect ratios. Using the formulated model, the effect of pore aspect ratio and size distribution on membrane performance was studied. It was found that increasing the pore aspect ratio improves membrane performance while increasing the width of the pore size distribution deteriorates the performance.

The effect of uniaxial stretching on membrane performance was also studied using a finite element model of a porous membrane in conjunction with the permeability-selectivity model. For the porous membrane modeled, improvement was observed in 15% and 30% uniaxial stretching cases. Further stretching deteriorated the membrane performance. The key factor in the deterioration of performance was found to be the width of the pore size distribution which became larger with stretch. Porosity dispersion was found to play a key role in pore size distribution of stretched membranes. Using the finite element model, it was determined that membranes with well-dispersed pores had less size distribution around the average value. This minimized the negative effect of pore size distribution on membrane performance. As a result, membranes with well-dispersed porosity had better performance improvement after stretching.

## Figures and Tables

**Table 1:** Material properties of dense PET used in finite element model.

**Property**	**Value**	
Bulk modulus, *K*	0.162 GPa	
Initial Shear modulus, G*_init_*	0.075 GPa	
Tangent modulus, *E_T_*	5.95 MPa	
**Viscoelasticity [15]**		
**Branch**	**Shear Modulus Ratio,** G*_vm_*/G*_init_*	**Relaxation Time (s)**
1	0.0402	10^−5^
2	0.0468	10^−4^
3	0.0572	10^−3^
4	0.1805	10^−2^
5	0.0487	10^−1^
6	0.0988	10^0^
7	0.0205	10^1^
8	0.1394	10^2^
9	0.0000	10^3^
10	0.1283	10^4^
11	0.0470	10^5^
12	0.1005	10^6^

**Table 2:** Size distribution parameters for stretched membranes.

Stretch	Avg. Major Axis Size, *a_avg_* (μm)	Avg. Minor Axis Size, *b_avg_* (μm)	Major Axis Size Distribution, σ/*a_avg_*	Major Axis Size Distribution, σ/*b_avg_*	Average Pore Aspect Ratio
Unstretched	0.097	0.097	0.060	0.060	1
15%	0.129	0.095	0.142	0.094	1.374
30%	0.162	0.087	0.191	0.156	1.866
40%	0.180	0.084	0.194	0.196	2.256
50%	0.199	0.079	0.201	0.256	2.732
